# Prevalence and correlates of HIV testing among adolescents 10–19 years in a post-conflict pastoralist community of Karamoja region, Uganda

**DOI:** 10.1186/s12889-018-5544-0

**Published:** 2018-05-10

**Authors:** Rogers N. Ssebunya, Rhoda K. Wanyenze, Leticia Namale, Heather Lukolyo, Grace P. Kisitu, Patricia Nahirya-Ntege, Adeodata Kekitiinwa

**Affiliations:** 1 Baylor College of Medicine Children’s Foundation, Mulago Hospital Complex, P.O. Box 72052, Clock Tower, Kampala, Uganda; 2 School of Public Health, Makerere University, College of Health Sciences, Kampala, Uganda

**Keywords:** Adolescents, HIV, HIV testing services, Uganda, Pastoralist communities

## Abstract

**Background:**

Adolescents are a priority group in HIV prevention and treatment. This study sought to determine the prevalence and correlates of HIV testing services (HTS) among adolescents in the pastoralist post-conflict area of Karamoja sub region, Uganda.

**Methods:**

A cross sectional study of 1439 adolescents aged 10–19 years, attending nine public health facilities in five of the seven districts of Karamoja, was conducted between August to September 2016. Adolescents were consecutively selected and interviewed using structured interviewer administered questionnaires. All respondents who had never tested for HIV were offered HTS. The main outcome was ever tested for HIV. Correlates of ever tested were analysed using multivariate logistic regression model.

**Results:**

Of the 1439 adolescents, 904 (62.8%) were females, 1203 (83.6%) were aged 15–19 years, 618 (43.0%) had attained primary education and 885 (61.5%) had ever had sex. Overall 1177 (81.8%) had ever tested and received HIV results. Older age (15–19 years) (adj.OR = 2.71, 95% CI: 1.85–3.96), secondary level education or higher (adj.OR = 2.33, 95% CI: 1.33–4.10), and ever had sex (adj.OR = 2.03, 95% CI: 1.42–2.90) were associated with higher odds of HIV testing. Of the 262 who had never tested, 169 (64.5%) accepted testing and 2.4% were HIV positive. Reasons for not accepting the test included fear of being tested and not ready for an HIV test because of perceived suffering HIV positive clients go through.

**Conclusion:**

Awareness of HIV status and uptake of HTS among adolescents in this hard-to-reach post-conflict region was high and close to the global UNAIDS target of 90%. However, the HIV prevalence of 2.4% among the non-testers who accepted to be tested was high and emphasises the need for targeted testing to reach the undiagnosed HIV infected adolescents in this region.

## Background

Adolescents and young people are a critical focus population in HIV prevention and treatment and particularly important in the attainment of the global targets towards elimination of HIV/AIDS by 2030. According to the 2016 UNICEF data, 670,000 out of 2.1 million (31.9%) new infections by 2015 were among young people aged 15–24 years— these included 250,000 infections among adolescents 15–19 years [1, 2]. New HIV infections among adolescents in sub Saharan Africa are not declining as quickly as among other age groups [3]. Adolescent females and young women aged 15–24 years are at a higher risk of HIV infection, contributing 25% of all new infections among adults in sub-Saharan Africa in 2015 [4]. Similar trends have been documented regarding AIDS-related mortality especially in sub-Saharan Africa where access to HIV testing, care and treatment services by young people is still a challenge [2, 3]. Every hour, 26 adolescents get infected, with close to 2 million living with HIV worldwide. This is compounded by low HIV-related knowledge—only 26% of the girls and 32% of the boys 15–19 years in sub-Saharan Africa know how HIV is transmitted and how it can be prevented [5]. Adolescents in hard to reach post-conflict and pastoralist communities where access to health care services including HIV testing services is limited are probably more disadvantaged. Such settings have also been linked to increased vulnerability to HIV and high-risk sexual behaviours [6–9].

Prevention of HIV transmission and acquisition among adolescents amidst multiple challenges including peer pressure, sense of invincibility and physiological changes calls for multi-dimensional interventions. HIV testing is a window for all HIV related care and treatment services and an essential step in achieving “the UNAIDS 90–90-90 targets”. However, globally only 35% of young people were aware of their HIV status in 2015 [10]. In sub-Saharan Africa, only 13% of the female and 9% of the male adolescents had ever tested for HIV and received their results in the last 12 months [1].

Awareness of HIV status and uptake of HIV Testing Services (HTS) varies considerably across different settings [11–13]. In a study in Malawi, HTS uptake ranged from 16.9% among the poor to 25.4% among individuals in the higher wealth quartile [14] while elsewhere uptake ranged from 27% to over 76.5% [15–18]. In Uganda, overall uptake of HTS among adolescents is less than 20%, however the reasons behind this low uptake are not well documented. Determinants of HTS uptake include age, education level, and willingness to disclose HIV results [19, 20]. Despite the variability in testing coverage, there is limited documentation of HIV prevalence and uptake of HTS among adolescents 10–19 years in remote, pastoralist, or post-conflict settings. A few of the studies that have elicited HTS uptake in post conflict communities in Uganda were among a small sample of youths 15–35 years [21], among children and adolescents (0-18 years) receiving mental health care services [22], and among refugees ≥20 years [23]. In a survey conducted in 2016 in Karamoja, HTS uptake among young people was 61.7% [24], however, the determinants of uptake were not highlighted. Additionally an understanding of the influence of contextual issues on HTS uptake for example; cultural transitions from isolation and fear from cattle raids to stability, urbanisation, and increased HIV/AIDS awareness provides an impetus for this study. The study therefore aimed to determine the prevalence and correlates of HIV testing among adolescents 10–19 years receiving primary health care services in a pastoralist and post-conflict region of Karamoja in Uganda. Adolescents who had never tested were offered HTS and the uptake as well as the reasons for refusal to test documented.

## Methods

### Study setting and population

Karamoja sub-region is located in the north eastern part of Uganda and is occupied by a pastoralist community that is dependent on animals for survival and security. In this setting, males do much of the animal rearing while females do the housework. This society has over the years had conflicts with other tribal neighbourhoods fighting for land, water and animals, a practice that has of recent stabilised when the government initiated a disarmament program and provided other logistical and humanitarian support in terms of food, shelter, health care and education. According to the 2011 Uganda AIDS indicator survey, general population HIV prevalence in Karamoja was 3.5% [25]—prevalence among female adolescents 15–24 years was 3.5% compared to 2.6% in their male peers [26]. The UNICEF annual report of 2013 showed Karamoja lagging behind compared to western Uganda and Acholi regions regarding access to HTS and antiretroviral therapy (ART) for prevention of mother to child transmission (PMTCT) [27].

### Study design, sample size and sampling

This was a cross-sectional survey involving 1439 adolescents (10–19 years) receiving primary health care services at the outpatient department (OPD) and maternal child health (MCH) clinics at public health facilities between August to September 2016. Bennett’s sample size formula [28] was used considering health facilities as the clusters and expected daily number of adolescents attending the facilities estimated at 140. Since the coverage of HIV testing among adolescents in this region was not known at the time of planning this study, a conservative prevalence of 50% and a design effect of 2.0 were used to yield a minimum sample size of 1375 participants from nine clusters. The highest volume facilities within the region—facilities that contributed 80% of all OPD attendance for adolescents in the previous year were sampled. The 80% mark was chosen to allow adequate representation of the districts and facilities within the region to be included in the study. The 9 facilities were located in five of the seven districts in the sub-region. The number of adolescents to be interviewed at each of these nine facilities was then determined using probability proportional to size (PPS), based on the numbers of adolescents who sought HTS in the prior quarter (April–June 2016) before data collection. Within each facility, adolescents were consecutively sampled until the required number per facility was obtained. All adolescents were approached for participation irrespective of their reason for coming to the facility.

### Data collection procedures

Ethical approval for this study was obtained from Makerere University School of Biomedical Sciences Higher Degree Research and Ethics Committee (SBSREC) and the Uganda National Council for Science and Technology (UNCST) before data collection. Additionally, permission was sought from district and health facility heads. After the selection, adolescents were screened for eligibility. Written informed parental or guardian consent and assent were obtained from adolescents < 18 years while those ≥18 years consented before enrolment into the study. Adolescents < 18 years who came to these facilities without a guardian or parent were advised to come back with a parent/guardian who could consent on their behalf. Parents/guardians and the adolescents were informed that the study included an assessment of knowledge and access to HIV services and that some would be offered an HIV test and tested if they accepted to do so. Respondents were interviewed by trained research assistants using semi-structure questionnaires and a modified HIV knowledge tool. All respondents were asked if they had ever tested for HIV and received their results. Those who had never tested where asked if they would like to be tested for HIV. Those who accepted were linked to the HTS sites within the health facilities.

Based on the institutional policies, all research assistants signed a confidentiality agreement before data collection to ensure confidentiality of respondent’s results. Those who refused to test for HIV were asked for the reasons, and these were documented through open ended questions. All questionnaires were checked by field supervisors, for quality control. On a daily basis, the completed questionnaires were collected by a regional supervisor who kept them locked in an office. Data was entered into an access database, cleaned from spreadsheets. The reasons for refusal to test for HIV among those who had never tested were also coded and entered into the database. Clean data was exported to Stata statistical software version 13.0 for analysis.

### Variables measurements

Data collected included; adolescents’ socio-demographic characteristics, HIV testing and receipt of results, knowledge of HIV prevention and transmission, knowledge of partner’s HIV status, engagement in high-risk sexual behaviours, history of having children, ever had sex, and use of substances or drugs. The main outcome in this study was “ever tested” for HIV which was coded as 1 for “Yes” and 0 for “No”. Independent variables included; socio-demographic characteristics like sex, age group, marital status, HIV knowledge (Yes = 1, No = 0), engaging in high-risk sexual behaviours (Yes = 1, No = 0), and use of drugs or other illicit substances (Yes = 1, No = 0). Sex was coded as (Female = 1, Male = 0), Age group coded as (15–19 years = 1 and 10-14 years = 0), Ever had sex coded as (Yes = 1, No = 0), Education level coded as (Nursery = 0, Primary = 1, ≥ secondary = 2). During testing for significant covariates; marital status was coded as; Never married = 0, Married/Cohabiting = 1 and Divorced/Separated = 2. HIV sero-status of their most recent partner was coded as (Yes = 1, No = 0), while the number of children was coded as (None = 0, 1–3 = 1). The HIV knowledge score was based on an aggregate score obtained by using a KQ-18 HIV questionnaire [29] modified to suit the cultural context for the study population. In line with other literature that considered mean and median scores for cut-off scores [30], participants in our study who scored above or equal to the median score of 11 were considered to have adequate HIV knowledge. Adolescents were considered to have engaged in high-risk sexual behaviours if they inconsistently used a condom and either had multiple sexual partners (two or more sexual partners) or having engaged in transactional sex in the last 6 months.

### Statistical analysis

Descriptive statistics for ever tested were presented as frequencies and percentages. A chi-square test was used to elicit associations between individual characteristics with HIV testing. Odds ratios were generated using a multivariate logistic regression model to elicit associations with HIV testing. We adjusted for a number of variables to include; sex of the adolescent, highest level of education attained, ever had sex, HIV knowledge, number of children the adolescent has ever had, knowing the HIV status of their sexual partners and high-risk sexual behaviours. We excluded marital status as an independent variable in the final model because of potential multi-collinearity with “ever had sex”. Variables with a *p*-value of 0.2 and below at bivariate analysis were entered into the multivariate models. Model parsimony was ensured by using the backward stepwise modelling and the likelihood ratio test between the full and restricted models. The model that yielded the highest variability in explaining the predicted variable (ever tested) was considered as the best fit. All analyses were conducted using STATA v.13 (College Station, TX).

## Results

### Baseline characteristics of study respondents

**Fig. 1:**
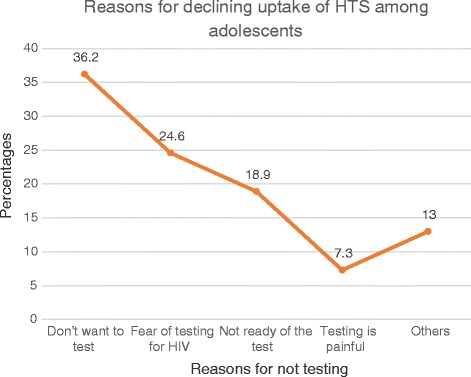
Reasons for declining taking an HIV test among adolescents 10–19 years

Overall, 1481 adolescents were approached for participation and 1443 (97.4%) were enrolled—four had incomplete data for key variables including the outcome variable and were excluded from analysis. Demographic and baseline characteristics of study respondents are summarized in Table 1. The majority of adolescents (904, 62.8%) were females, aged 15–19 years (1203, 83.6%) and were married or cohabiting (526, 36.6%). The median age of study participants was 18.0 (iqr = 3) for both males and females. More than half of the adolescents (737, 51.2%) were in school and nearly 82% (1177) of the adolescents had ever tested for HIV and received results by the time of the interview, of whom nine (0.8%) were positive. Of the 262 adolescents who had never tested for HIV, 169 (64.5%) accepted testing, of whom four (2.4%) were HIV positive. Thus overall, 13 of the 1346 adolescents who tested (1.0%) were HIV positive. Figure 1 shows key reasons why adolescents who had never tested refused to test. Among the 93 adolescents who declined testing, for the largest percentage (*n* = 25, 36.2%) did not offer any specific reason—they just did not want to test. Others feared testing (*n* = 17, 24.6%) and perceived testing to be painful (*n* = 7, 7.3%) or did not feel ready for the test (*n* = 13, 18.8%). Of the 71 sexually active adolescents who declined an HIV test, only 2 (2.8%) had engaged in high-risk sexual behaviors. A small proportion (1.4%) of adolescents reported to have ever used drugs or substances like marijuana, kuber and tobacco smoking.

**Table 1:** Baseline characteristics of study respondents

Variable, *N* = 1439		N	Percent (%)
Age	10–14	236	16.4
	15–19	1203	83.6
Sex	Males	535	37.2
	Females	904	62.8
Marital status	Never married	901	62.6
	Married/cohabiting	526	36.6
	Divorced/Separated	12	0.8
Highest level of education	No education & Nursery/ECD	354	24.6
	Primary/ABEK^a^	618	43.0
	≥ Secondary	467	32.5
Occupation	Student/Pupil	737	51.2
	Cattle keeper	83	5.8
	Other & None	619	43.0
Ever had children	None	1079	75.0
	1–3 children	360	25.0
Ever tested for HIV	Yes	1177	81.8
	No	262	18.2
Uptake of HTS, *n* = 262	Yes	169	64.5
	No	93	35.5
HIV prevalence	Positive	4	2.4
	Negative	165	97.6
Use of drugs/Substances	Yes	20	1.4
	No	1419	98.6

^a^*ABEK* Alternative Basic Education for Karamoja, *ECD* Early Childhood Development

### Adolescent characteristics by HIV testing status

Results in Table 2 show that a much higher percentage of older adolescents 15–19 years (1051, 89.3%) ever tested for HIV and received results compared to their younger counterparts (126, 10.7%). Results further indicate that equal proportions (49.3% vs 50.7%) had ever tested for HIV among those with and without adequate HIV knowledge. A stratified analysis among sexually active adolescents (*n* = 885), 85 (9.6%) had never been tested for HIV. Additionally, of the 301 adolescents who never knew the sero-status of their most recent sexual partners, 60 (19.9%) had never tested for HIV. Of the 101 adolescents who engaged in high-risk sexual behaviours, 15 (14.9%) had never tested for HIV.

**Table 2:** Relationship between HIV testing and individual characteristics of adolescents

Variable		Ever tested		*p*-value
		Yes, *n* = 1177	No, *n* = 262	
Sex	Male	414 (35.2)	121 (46.2)	0.001*
	Female	763 (64.8)	141 (53.8)	
Age group	10–14	126 (10.7)	110 (42.0)	0.001*
	15–19	1051 (89.3)	152 (58.0)	
Adequate HIV knowledge	Yes	580 (49.3)	91 (34.7)	0.001*
	No	597 (50.7)	171 (65.3)	
Education status	No Education/Nursery	288 (24.5)	66 (25.2)	0.001*
	Primary/ECD	466 (39.6)	152 (58.0)	
	≥ Secondary	423 (35.9)	44 (16.8)	
Ever had sex	No	377 (32.0)	177 (67.6)	0.001*
	Yes	800 (68.0)	85 (32.4)	
Use of drugs or substances	No	1158 (98.4)	261 (99.6)	0.123
	Yes	19 (1.6)	1 (0.4)	
Knows partners sero-status^a^	Yes	559 (69.9)	25 (29.4)	0.001*
	No	241 (30.1)	60 (70.6)	
High-risk sexual behaviours^a^	Yes	86 (10.8)	15 (17.7)	0.057
	No	714 (89.2)	70 (82.3)	

^a^Among those who have ever had sex (*n* = 885)

*significance at *p* < 0.05

### Correlates for ever tested among adolescents

Table 3 highlights three statistically significant associations with HIV testing among adolescents i.e. age of the adolescent, level of education and ever had sex. Older adolescents aged 15–19 years had higher odds (adj.OR = 2.71, 95% CI: 1.85, 3.96) of HIV testing compared to their younger counterparts aged 10–14 years. Attaining secondary or higher level of education was associated with increased odds of HIV testing compared to those who had no education at all; (adj.OR = 2.33, 95% CI: 1.33, 4.10). Additionally adolescents who had ever had sex were more likely to have been tested compared to those that had never; (adj.OR = 2.03, 95% CI: 1.42, 2.90). Further still, having adequate level of HIV knowledge, sex, and the parity of the adolescents i.e. whether they have ever had children or not, and engagement in high-risk sexual behaviours were not statistically significant contributors to ever been tested for HIV.

**Table 3:** Correlates of Ever tested for HIV among study respondents

Variable		Ever tested, *n* = 1177	Percent (%)	Unadj.OR	95% CI	adj.OR	95% CI	*p*-value
Sex	Male	414	35.2	1.0 (ref)				
	Female	763	64.8	1.58	1.21–2.07	1.23	0.89–1.71	0.217
Age group	10–14	126	10.7	1.0 (ref)				
	15–19	1051	89.3	6.04	4.44–8.21	2.71	1.85–3.96	0.001*
Adequate HIV knowledge	No	597	50.7	1.0 (ref)				
	Yes	580	49.3	1.83	1.38–2.41	1.33	0.98–1.81	0.07
Education status	No Education/ Nursery	288	24.5	1.0 (ref)				
	Primary/ ECD	466	39.6	0.70	0.51–0.97	1.14	0.91–2.20	0.126
	≥ Secondary	423	35.9	2.20	1.46–3.32	2.33	1.33–4.10	0.003*
Ever had Sex	No	377	32.0	1.0 (ref)				
	Yes	800	68.0	4.42	3.32–5.88	2.03	1.42–2.90	0.001*
Use of drugs or substances	No	1158	98.4	1.0 (ref)				
	Yes	19	1.6	4.28	0.57–32.1	2.19	0.27–17.4	0.460

*Significance at *p* < 0.05

## Discussion

This cross-sectional study aimed to assess the prevalence and correlates of HIV testing among adolescents in a post-conflict, pastoralist setting. Over 80% of the adolescents had ever tested prior to the survey, close to the UNAIDS 90% target. Overall HIV prevalence among adolescents who tested before and during the study was 1.0% – which is lower than the national HIV prevalence among young males 15–24 years (1.9%) and females (3.8%) [31]. However, among those who had previously not tested, the prevalence was 2.4%—although these numbers were small, this highlights the need for targeted adolescent services to reach the remaining undiagnosed HIV infections as we approach the first 90% target.

Our study shows much higher HIV testing among adolescents than the 2016 Uganda demographic health survey (UDHS) [32] and in other studies elsewhere [21, 33]. This community was ravaged by tribal conflicts for over a decade, however, the post-conflict phase and stability led to prioritization of the region and more focused interventions from development partners and implementers, which may explain the much higher coverage and uptake of testing among adolescents in this region [34]. However, despite the high uptake, there is need to sustain these efforts given the significant proportion of adolescents who tested positive among those who had never tested.

Age, level of education, and ever had sex were significantly associated with higher levels of HIV testing. Older adolescents aged 15–19 years had higher odds of HIV testing. In this setting, older adolescents especially boys, take on the role of warriors and looking after cattle and are thus more mobile and probably more likely to access health services. Older adolescents also have more autonomy and decision making powers including health seeking and are also more likely to be sexually active due to the culture of early marriages in this community. This trend has also been documented in other studies [18, 35] and highlights the need to mitigate the barriers in accessing HIV testing services in the younger age groups. Whereas the younger adolescents may not be sexually active yet, they should also be targeted for testing given the potential for perinatally transmitted HIV infection [36, 37].

Adolescents who have ever had sex were more likely to have tested for HIV than those who had not. This result could indicate a higher desire among sexually active adolescents to know their sero-status following sexual exposure or prior to sexual encounters. Evidence shows a higher likelihood of HIV testing among individuals who have been exposed to risk behaviors including unprotected sexual encounters and sexually transmitted infections [38–40]. This finding also underscores the need to also target adolescents who have never had any sexual encounter but could be infected due to perinatal exposure.

It is not surprising that adolescents with higher education were more likely to have been tested for HIV since several service providers offer HTS outreaches and door-to-door HIV testing in communities including schools [41, 42].

The high level of stigma towards HIV/AIDS that has been reported in this setting may explain the > 30% refusal to test among adolescents who had never tested [21, 43]. Adolescent tailored communication strategies including mitigating of stigma in conflict and mobile communities could increase uptake of HIV services.

## Limitations

Our study had several limitations. First the study was facility based and thus excluded those that might have barriers to accessing health facilities in general—this could over-estimate the uptake of HIV testing. Secondly adolescents who tested early in their life but remained sexually active with partners of unknown HIV status were not retested in this study. Finally, adolescents whose guardians did not consent were excluded—however this was a very small number (< 12) among the thousands interviewed and could thus not have significantly influenced the findings. We recommend further research to explore mechanisms of enhancing efficiency for HTS by improving the targeting of HIV testing to adolescents at high risk of HIV infection.

## Conclusion

Awareness of HIV status and uptake of HTS among adolescents in this hard-to-reach post-conflict pastoralist region was high and close to the global UNAIDS target of the first 90. However, the HIV prevalence of 2.4% among those that had never tested and accepted to be tested highlights the need for targeted and risk-based testing to reach the undiagnosed HIV-infected adolescents.

## Abbreviations

ANC: Antenatal care
HIV: Human immunodeficiency virus
HTS: HIV testing Services
MCH: Maternal Child Health
OPD: Out Patient Department
SMC: Safe Medical male circumcision
UNAIDS: Joint United Nations Programme on HIV/AIDS
